# Vertebral Arteriovenous Fistula: An Unwelcome Thrill

**DOI:** 10.1155/2017/8386459

**Published:** 2017-04-05

**Authors:** Matthew K. Edwards, Erica N. Christenson, Brian M. Corliss, Adam J. Polifka, Brandon R. Allen

**Affiliations:** ^1^Department of Emergency Medicine, University of Florida Health, Gainesville, FL, USA; ^2^Department of Neurosurgery, University of Florida Health, Gainesville, FL, USA

## Abstract

Cervical vertebral AV fistulae are uncommon vascular lesions involving abnormal communication between the extradural vertebral artery and surrounding venous structures. We examine the case of a female evaluated in the emergency department with a vertebral AV fistula presenting classically as pulsatile tinnitus and later successfully treated with standard endovascular techniques. A discussion on the etiology, pathophysiology, and management of vertebral AV fistulae follows.

## 1. Case Presentation

A 32-year-old female was evaluated in our emergency department for the chief complaint of right-sided pulsatile tinnitus. The patient stated that her tinnitus had begun six months prior and was initially low in amplitude and only noticeable when using a stethoscope in her work as a registered nurse. However, her tinnitus became progressively louder over the two months before presentation, thus prompting her to seek medical evaluation. She noted several associated symptoms, including persistent headache, an irritating audible “whooshing” sound, and more recently a transient globus sensation. She denied any history of significant head or neck trauma. Her medical history was notable only for unchanged, chronic neck pain due to preexisting cervical spondylosis and she reported no known family history of hereditary diseases. She took low-dose aspirin daily and was undergoing hormonal therapy in preparation for in vitro fertilization, though she did not specify the medications involved in treatment.

**Figure 1:**
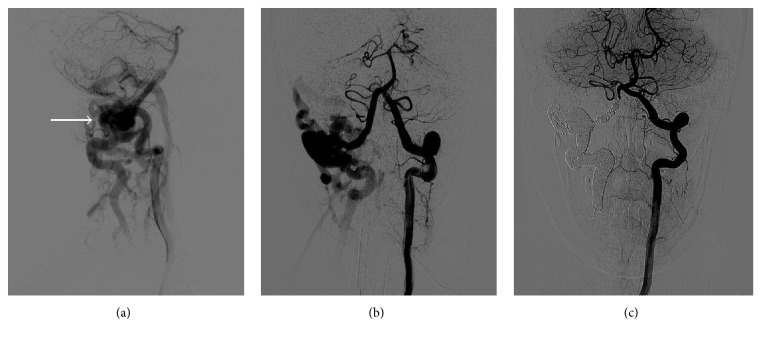
(a) and (b) Preoperative vertebral angiogram right anterior oblique (a) and anteroposterior (b) views showing an arteriovenous fistula (indicated by arrow) at the level of C1, with no obstruction of the left vertebral artery. (c) Postoperative vertebral angiogram anteroposterior view shows no evidence of fistula.

On examination, a palpable thrill and loud bruit were evident over the right neck below the angle of the jaw. The patient's vital signs were within normal limits and she was neurologically intact. The ultrasound and computed tomography angiogram (CTA) of the neck demonstrated abnormal vertebral artery anatomy bilaterally, with a dilated and tortuous right vertebral artery and a smaller left vertebral artery entering the cervical foramen at C4. The CTA also demonstrated multiple dilated and serpiginous vessels surrounding the right vertebral artery at the level of C1 and opacification of the right internal jugular vein and epidural venous plexus on the right, suggesting a potential arteriovenous fistula (AVF) involving the right vertebral artery (Figures 1(a) and 1(b)). The Department of Neurosurgery was consulted for further evaluation and treatment.

The patient was admitted to the neurosurgery service and diagnostic angiography the next day confirmed the presence of a fistula. On day two of admission, she underwent balloon test occlusion followed by endovascular sacrifice of the right vertebral artery and coil embolization of the fistulous communication (Figure 1(c)). She tolerated the procedure well and had immediate resolution of her pulsatile tinnitus, though she had some difficulty with postprocedural pain. On day five of admission, once established on an appropriate regimen of oral analgesics, she was discharged home in good health. At follow-up, the patient reported a right-sided headache originating at the occiput and radiating forward, but she remained neurologically intact, and angiography three months after operation confirmed successful obliteration of the fistula.

## 2. Discussion

Cervical vertebral AV fistulae are uncommon vascular lesions involving abnormal communication between the extradural vertebral artery and surrounding venous structures, including the epidural venous plexus and/or jugular venous system. Due to the rarity of its clinical presentation, the morbidity and mortality of this disease are not well established but are thought to be high if left untreated. Vertebral AVFs are often traumatic in origin, resulting from penetrating neck wounds, blunt trauma with vertebral fracture, and iatrogenic injuries of the neck including those from jugular vein catheterization [1–3]. Infrequently, vertebral AVFs arise through atraumatic processes, either congenitally or spontaneously. Approximately one-third of those that are spontaneous occur in patients with underlying genetic disorders, like neurofibromatosis type 1, or connective tissue disorders, such as Ehler-Danlos syndrome [4]. Vertebral AVFs most frequently present as pulsatile bruits and tinnitus, a result of turbulent blood flow within the aberrant arteriovenous connection [4, 5]. Less common manifestations include vertigo, neurological deficits, and neck pain, though up to 30% of vertebral AVFs may be asymptomatic at diagnosis [6, 7].

Our patient's vertebral AVF was typical in its symptomatic presentation, including intractable tinnitus and pulsatile bruit. She further reported a chronic history of neck pain, seemingly unaffected by the formation of her fistula. The patient attributed her ongoing neck pain with a prior diagnosis of “military neck,” a term predominantly used by the chiropractic profession. Though she did not associate onset of her symptoms with any known physical trauma, invasive medical procedure, or pathology, numerous prior cases of vertebral AVFs report induction by chiropractic manipulation [8, 9]. It is therefore plausible that our patient's fistula was caused by unreported chiropractic manipulation, although this patient's fistula occurred in the distal cervical portion of her vertebral artery, where previous studies have determined that spontaneous vertebral AVFs form most commonly [6, 10].

Even when asymptomatic, treatment of vertebral AVFs is generally regarded as appropriate because worsening neurological compromise may develop if the fistula grows. Delay of treatment also allows time for the fistula to recruit additional feeding vessels that may make future treatment more difficult. If left untreated, these fistulae can produce symptoms of vertebrobasilar insufficiency via vascular steal phenomena, aneurysm formation with subsequent thromboembolism due to abnormal flow patterns, compressive myeloradiculopathy due to progressive engorgement of the cervical epidural veins, or catastrophic intramedullary hemorrhage due to intramedullary venous hypertension [4]. Historically, ligation was the only available intervention, though compared to current techniques it is considered more invasive and challenging to perform given the artery course through the cervical vertebrae [4]. Endovascular methods, including the use of detachable balloons, liquid agents, coils, and stent grafts, have greatly enhanced the success and safety of treating AVFs. Regardless of treatment modality, artery sacrifice risks ischemia if the contralateral blood supply is insufficient or becomes compromised at a later time [11]. Placement of stent grafts may be preferred in these cases because it preserves flow through the treated vessel, though potential in-stent stenosis and incomplete closure due to inadequate vessel wall apposition present risk with this technique as well [6].

Vertebral artery sacrifice with coil embolization, the placement of small metal coils to occlude vascular flow through the fistula, was chosen as the treatment modality for this patient after she successfully tolerated balloon test occlusion of the involved vertebral artery. Balloon test occlusion entails the use of an endovascular balloon catheter to temporarily halt blood flow through the vessel in question. During occlusion, hypotension is induced and the patient's neurological status is monitored either while the patient is awake or, as was done in this case, through electrophysiologic monitoring on an anesthetized patient. If the patient does not experience symptoms of cerebral ischemia, the collateral flow to the brain from other vessels is assumed to be sufficient to accommodate the occluded vessel.

Our patient is also notable for her atypical artery course, with her left vertebral artery entering the cervical foramen at C4. Deviations from the typical course of the vertebral artery through the C6 transverse foramen are rare, with Bruneau et al. [12] finding entry at level of C4 in only 1.0% of their 500 study subjects. While typically asymptomatic, alterations in artery course are associated with an increased risk of additional vascular anomalies, including aneurysms and angiomas, and pose risk for surgical complication [12, 13].

Because the vertebral AV fistula is rarely seen in the emergency department, this case is presented to improve awareness of this disease's clinical manifestation and management. Emergency physicians should consider this diagnosis in patients complaining of pulsatile tinnitus and demonstrating loud bruit and palpable thrill on exam.
