# Corrigendum to “Vertebral Arteriovenous Fistula: An Unwelcome Thrill”

**DOI:** 10.1155/2018/7514784

**Published:** 2018-08-09

**Authors:** Matthew K. Edwards, Erica N. Christenson, Brian M. Corliss, Adam J. Polifka, Brandon R. Allen

**Affiliations:** ^1^Department of Emergency Medicine, University of Florida Health, Gainesville, FL, USA; ^2^Department of Neurosurgery, University of Florida Health, Gainesville, FL, USA

In the article titled “Vertebral Arteriovenous Fistula: An Unwelcome Thrill” [1], there was an editorial error, which was indicated in the Letter to the Editor by Foreman et al. [2]. The authors apologize for mistakenly including the case report by Foreman et al. [3] as an example of vertebral AV fistulae potentially induced by chiropractic manipulation. As they correctly observed, the intracranial location of their patient's AV fistula argues against this etiology. Instead, the authors intended to cite Briganti et al. who reported on a patient with an extradural AV fistula that formed after spinal manipulation [4].

The authors originally included the publication by Foreman et al. in their discussion on AV fistulas not associated with chiropractic manipulation, which was later removed during editing. Although there are many studies on patients suffering vertebrobasilar stroke and arterial dissection following chiropractic neck manipulation, temporal association does not necessarily equate causation. The authors praise Foreman et al. for their case report, which illustrates the danger of etiological assumption and thank them for drawing attention to the need for more rigorous investigation into causes of AV fistula formation.
